# Older Age, Early Symptoms and Physical Function are Associated with the Severity of Late Symptom Clusters for Men Undergoing Radiotherapy for Prostate Cancer

**DOI:** 10.1016/j.clon.2018.01.016

**Published:** 2018-06

**Authors:** A. Lemanska, D.P. Dearnaley, R. Jena, M.R. Sydes, S. Faithfull

**Affiliations:** ∗ School of Health Sciences, Faculty of Health and Medical Sciences, University of Surrey, Guildford, UK; † Institute of Cancer Research and Royal Marsden NHS Trust, London, UK; ‡ Cambridge University Hospitals, Addenbrookes Hospital, Cambridge, UK; § MRC Clinical Trials Unit at UCL, Institute of Clinical Trials and Methodology, London, UK

**Keywords:** Acute symptoms, late symptoms, PROs, prostate cancer, radiotherapy, survivorship, symptom clusters

## Abstract

**Aims:**

To identify symptom clusters and predisposing factors associated with long-term symptoms and health-related quality of life after radiotherapy in men with prostate cancer.

**Materials and methods:**

Patient-reported outcomes (PROs) data from the Medical Research Council RT01 radiotherapy with neoadjuvant androgen deprivation therapy trial of 843 patients were used. PROs were collected over 5 years with the University of California, Los Angeles Prostate Cancer Index (UCLA-PCI) and the 36 item Short-Form Health Survey (SF-36). Symptom clusters were explored using hierarchical cluster analysis. The association of treatment dose, baseline patient characteristics and early symptom clusters with the change in severity of PROs over 3 years was investigated with multivariate linear mixed effects models.

**Results:**

Seven symptom clusters of three or more symptoms were identified. The clusters were stable over time. The longitudinal profiles of symptom clusters showed the onset of acute symptoms during treatment for all symptom clusters and significant recovery by 6 months. Some clusters, such as physical health and sexual function, were adversely affected more than others by androgen deprivation therapy, and were less likely to return to pretreatment levels over time. Older age was significantly associated with decreased long-term physical function, physical health and sexual function (*P* < 0.001). Both baseline and acute symptom clusters were significant antecedents for impaired function and health-related quality of life at 3 years.

**Conclusions:**

Men with poorer physical function and health before or during treatment were more likely to report poorer PROs at year 3. Early assessment using PROs and lifestyle interventions should be used to identify those with higher needs and provide targeted rehabilitation and symptom management.

## Highlights

• Short- and long-term (up to 5 years) effects of radiotherapy with ADT were shown using PROs.
• Both baseline and acute symptoms were antecedents for impaired late QOL and function.
• Older age and poorer general health were associated with poorer outcomes at 3 years.
• Early assessment using PROs should be used in treatment planning and targeted prehabilitation.

## Introduction

Prostate cancer (PCa) survival has improved significantly over the last decade. More than 84% of men now survive 10 years or more in the UK [1] and the number of survivors is growing by 3% every year [2]. Globally, more than 1.1 million cases of PCa were recorded in 2012. This constitutes 8% of all cancers and 15% of cancers in men, making PCa the second most common cancer in men [3]. With increasing survival, cancer-related symptoms and treatment-related toxicity can affect men's long-term health-related quality of life (HRQOL) [4]. Common side-effects after prostate radiotherapy include decreased urinary, bowel and sexual functions and these affect supportive care needs of men [5]. Population-based studies of long-term functional outcomes after PCa suggest that at 12 years from treatment, 87% of men will have erectile dysfunction or sexual inactivity, 20% urinary incontinence and 14% bowel problems [6]. These figures are substantially lower in men without cancer and of a similar age (62, 6 and 7%, respectively). Androgen deprivation therapy (ADT) is commonly used with radical radiotherapy for intermediate- or high-risk PCa. It allows for better long-term PCa control than with radiotherapy alone. However, it can add fatigue, hot flushes or muscle and bone loss to the spectrum of expected side-effects [7], [8].

The prevalence of long-term side-effects from radiotherapy depends on many factors. Treatment factors, such as total dose or fractionation schedule, and individual factors, such as age, comorbidities or medical history (e.g. previous surgery), can all affect late toxicity [9]. Patients undergoing radiotherapy experience groups of symptoms called clusters in response to cancer or treatment. Symptom clusters were first defined in cancer by Miaskowski *et al.* in 2004 [10]. They are groups of symptoms with similar prevalence rates and related by a common aetiology or by influencing similar patient outcomes. Since then the concept has served as a basis for the assessment and management of multiple symptoms. There has been substantive research into defining and identifying symptom clusters in a variety of cancers and oncology treatments [11]. Fatigue, insomnia, pain and depression constitute the most prevalent symptom cluster in cancer [12]. Synergy of symptoms in clusters has been studied and the effect on HRQOL, functional status and survival has been described [13], [14].

In PCa there have been only two studies examining symptom clusters related to the early stage disease or its treatment [15], [16]. Maliski *et al.* [15] found that fatigue and emotional distress were common in this group of patients and they clustered together with urinary, bowel and sexual symptoms. Capp *et al.* [16] only explored rectal symptoms in their longitudinal study. They found that symptom clusters were stable over time and that rectal urgency and pain were the core drives of symptom clustering [16]. A different longitudinal study of Knapp *et al.* [17] explored trajectories and predictors of radiotherapy-related PCa symptoms over 25 weeks. They found that pain, fatigue, insomnia and diarrhoea were highly prevalent and related to symptom distress. Only a limited number of longitudinal studies are available in PCa and none report symptom clusters over a 5 year trajectory. A limitation of studies that analyse radiotherapy-related data in a cross-sectional manner is that the effect of baseline symptoms on time trajectory is not considered and it may be attributed to radiation toxicity.

It is important to consider baseline symptoms when assessing treatment-related side-effects. This is because both baseline and acute symptoms have been found to be a precursor of late symptoms. This has been termed as consequential late effects [18], [19]. In addition, treatment factors (ADT, radiotherapy dose or fractionation) have been found to directly affect acute and late symptoms [20]. However, the research into treatment side-effects is now complemented by the evidence of an indirect effect of patient characteristics, such as age, functional status or comorbidities [21], [22], [23]. Despite the increasing interest and growing body of evidence, identification and prediction of long-term symptom clusters in PCa, to establish links between symptoms and the role of other contributing factors, remains a challenge. Men with PCa could benefit from this through targeted symptom management approaches that address multiple symptoms and risk factors.

## Materials and Methods

### Study Design and Research Questions

Longitudinal profiles of patient-reported outcomes (PROs) were explored and symptom clusters investigated using well-established symptom clustering methodologies [24], [25]. Patterns in PROs data, reported up to 5 years after treatment by men in the Medical Research Council (MRC) RT01 clinical trial were investigated to study: (i) which PROs were associated and formed symptom clusters, to investigate what symptom clusters are experienced by men with PCa during and after radiotherapy; (ii) how symptom clusters change over time, to investigate the effect of ADT and radiotherapy treatment on the trajectory of symptom clusters during the 5 years of follow-up; (3) the association of treatment, demographics, medical history, i.e. comorbidities, and baseline and acute symptom clusters with the change in symptom clusters over 3 years, to investigate potential risk factors contributing to late symptom clusters. Secondary data analysis was agreed by the MRC RT01 trial team and received appropriate ethical approval.

### Dataset and Patients

We used the MRC RT01 trial (ISRCTN47772397), which is a dataset of 843 patients [20], [26]. It was a UK-led, multicentre, randomised controlled trial that investigated standard (64 Gy/32 fractions) versus escalated (74 Gy/37 fractions) conformal radiotherapy with neoadjuvant ADT for patients with localised PCa. Eligible men had histologically confirmed PCa and prostate-specific antigen <50 ng/ml, no previous PCa treatment and no significant medical history that excluded them from radical radiotherapy. Men were followed in the study for up to 5 years. PROs were recorded with the University of California, Los Angeles Prostate Cancer Index (UCLA-PCI), which also included the RAND 36 item Short-Form Health Survey (SF-36). A detailed study design, patient eligibility criteria and main results have been published [27], [28].

### Measurements and Outcome Variables

Patient baseline characteristics are presented in Table 1. Information includes treatment group, age, tumour stage, Gleason score, prostate-specific antigen, comorbid conditions (diabetes, hypertension, inflammatory bowel, haemorrhoids) and medical history, such as type of biopsy, previous pelvic surgery or previous transurethral resection of the prostate. PROs were collected before ADT (baseline), during radiotherapy (acute) and until 5 years after radiotherapy (long-term). PROs included 20 items of the UCLA-PCI [29] measuring function and symptom bother in the three PCa primary concern areas (urinary, bowel and sexual scales); and 36 items of the SF-36 measured HRQOL arranged in eight multi-item scales (physical functioning, role limitations due to physical health, role limitations due to emotional problems, bodily pain, vitality, mental health, social functioning and general health) [30]. In total, 56 PROs recorded on a Likert scale were included in the secondary data analysis. They were collected by patient self-report at the following 10 time points: pre-ADT, preradiotherapy, at the end of radiotherapy (at week 10 after the start of radiotherapy), every 6 months until year 2 (months 6, 12, 18 and year 2) and then yearly for up to 5 years (years 3, 4 and 5 after the start of radiotherapy).

**Table 1:** Medical Research Council RT01 study: patient baseline characteristics (*n* = 843)

Characteristic	Mean (standard deviation)	*n*	%
Radiotherapy treatment dose			
Standard		421	50
Escalated		422	50
Age (years)	67 (6)		
Stage			
T1		209	25
T2		475	56
T3		147	17
Missing		12	1
Gleason score			
2–4		70	8
5–6		411	49
7		191	23
8–10		96	11
Missing		75	9
PSA (ng/ml)	15.4 (10)		
Missing		6	1
Diabetes		55	6
Missing		8	1
Hypertension		252	30
Missing		8	1
Inflammatory bowel or any diverticular disease		36	4
Missing		12	1
Haemorrhoids in past 12 months		89	11
Missing		18	2
Type of biopsy			
Transrectal		715	85
TURP		102	12
Other		19	2
Missing		7	1
Previous pelvic surgery		48	6
Missing		13	1
Previous TURP		100	12
Missing		14	2

PSA, prostate-specific antigen; TURP, transurethral resection of the prostate.

### Missing Data and Data Pretreatment

The number of data missing in patient baseline characteristics is detailed in Table 1. The number of PRO questionnaires completed at each time point is detailed in Table 2. These missing data were not imputed. The number of single questions left unanswered in completed questionnaires (intermittent missing data) varied from 0.8% for feeling tired (SF-36 vitality scale) to 13.4% for urinary leak interfering with sex (UCLA-PCI sexual function scale). Intermittent missing data were treated with multiple imputation, rather than using complete case analysis, to minimise the risk of biased results and to preserve the sample size [31]. Five imputations are usually sufficient, but seven imputed datasets were created to further reduce the uncertainty in the prediction of missing values process [32]. Variables were rescaled to a 0 to 100 scale for consistency (0 representing the worst outcome and 100 representing the best possible outcome), including reversing negatively worded questions, as recommended by the scoring manual [33].

**Table 2:** Results of symptom clustering. Items collected with the University of California, Los Angeles Prostate Cancer Index (UCLA-PCI) and the 36 item Short-Form Health Survey (SF-36) that belong to a cluster in a given point in time are marked with an ‘x’ in the table. Core cluster symptoms (present in clusters across time) are marked in bold. Symptoms never present in the symptom cluster are marked with an asterisk. Seven symptom clusters were identified: physical function, physical health, emotional health, vitality, illness perception, urinary function and sexual function

Tool	UCLA-PCI and SF-36 scales and items	Point in time									
		Pre-ADT (*n* = 578)	Pre-RT (*n* = 757)	Week 10 (*n* = 738)	Month 6 (*n* = 712)	Month 12 (*n* = 689)	Month 18 (*n* = 655)	Year 2 (*n* = 645)	Year 3 (*n* = 594)	Year 4 (*n* = 515)	Year 5 (*n* = 425)
**SF-36**	**Physical functioning**	**Physical function cluster**									
	Vigorous activities*										
	Moderate activities	x				x	x				
	Lifting/carrying	x				x	x				
	Climbing several flights of stairs	x	x		x		x	x	x	x	X
	Climbing one flight of stairs	x	x			x					
	**Walking one mile**	x	x	x	x	x	x	x	x	x	x
	**Walking several blocks**	x	x	x	x	x	x	x	x	x	x
	Walking one block	x	x	x		x	x				
	Bending/kneeling*										
	Bathing/dressing*										
**SF-36**	**Role limitations due to physical health**	**Physical health cluster**									
	Cut down on activities	x	x	x	x	x	x	x	x	x	
	**Accomplished less**	x	x	x	x	x	x	x	x	x	x
	**Limited in kind of work**	x	x	x	x	x	x	x	x	x	x
	**Difficulty working**	x	x	x	x	x	x	x	x	x	x
**SF-36**	**Role limitations due to emotional problems**	**Emotional health cluster**									
	**Cut down on activities**	x	x	x	x	x	x	x	x	x	x
	**Accomplished less**	x	x	x	x	x	x	x	x	x	x
	Did not work as carefully	x	x	x	x	x	x	x			x
**SF-36**	**Vitality**	**Vitality cluster**									
	Full of life			x	x	x	x		x	x	
	A lot of energy			x	x	x	x		x	x	
	Worn out			x	x	x	x		x	x	
	Feeling tired			x	x	x	x		x	x	
**SF-36**	**General health**	**Illness perception cluster**									
	Get sick easier*										
	**As healthy as anyone**	x	x	x	x	x	x	x	x	x	x
	**Health is excellent**	x	x	x	x	x	x	x	x	x	x
	**Health in general**	x	x	x	x	x	x	x	x	x	x
	Health get worse*										
**UCLA-PCI**	**Urinary function**	**Urinary function cluster**									
	**Urinary leak**	x	x	x	x	x	x	x	x	x	x
	**Urinary control**	x	x	x	x	x	x	x	x	x	x
	**Dripping/wetting**	x	x	x	x	x	x	x	x	x	x
	Number of pads or diapers*										
	Urinary leak interfering with sex*										
**UCLA-PCI**	**Sexual function**	**Sexual function cluster**									
	Sexual desire	x			x			x		x	x
	**Erection ability**	x	x	x	x	x	x	x	x	x	x
	**Orgasm ability**	x	x	x	x	x	x	x	x	x	x
	**Quality of erections**	x	x	x	x	x	x	x	x	x	x
	**Frequency of erections**	x	x	x	x	x	x	x	x	x	x
	**Sexual function overall**	x	x	x	x	x	x	x	x	x	x
	Awakened with erections				x	x	x		x	x	x
	Intercourse*										

ADT, androgen deprivation therapy; RT, radiotherapy.

### Symptom Clusters Analysis

Symptom clustering was carried out at each point in time on completed PROs questionnaires (intermittent missing data imputed with multiple imputation). Similarity between symptoms was measured with Spearman's rho correlation coefficient (*r*_s_). To obtain pooled correlation results from the seven imputed datasets, composite correlations were calculated using Fisher's *z* transformation [34]. Clustering between PROs was identified using hierarchical cluster analysis with the average linkage method of cluster agglomeration. Symptom clusters were determined at a cut-off correlation value of >0.60 [25].

### Multivariate Linear Mixed Effects Regression Analysis

Multivariate linear mixed effects modelling was used to calculate the contribution of early symptoms and other potential risk factors, such as treatment, age, medical history and other symptom clusters, to the change in symptom clusters over time. Composite scores of symptom clusters at three time points were used in longitudinal modelling: baseline (pre-ADT or pre-RT if pre-ADT was not collected), acute (week 10) and late (year 3). Three years after radiotherapy has been used as an end point because it has been shown to be an important point in time for the recognisable development of late radiotherapy-related symptoms [26], [35].

Longitudinal profiles of symptom clusters were the dependent variables for the models. Independent variables that were investigated included baseline age, comorbidities and medical history, as well as other symptom clusters that were included as fixed effects. Radiotherapy dose, time and individual patient variation were included in the models as random effects. Independent variables with preliminary significant associations of *P* < 0.05 were retained in the final regression models. A statistical significance level of *P* < 0.01 rather than 0.05 was used to account for multiple statistical tests that were carried out. The statistical significance was estimated using the likelihood ratio test [36]. The analysis was performed with R version 3.0.2 (R Foundation for Statistical Computing, Vienna, Austria).

## Results

### Symptom Clusters

Seven symptom clusters of three or more associated symptoms were identified and named as: physical function; physical health; emotional health; vitality; illness perception; urinary function; and sexual function. The results of symptom clustering and the number of questionnaires collected at each time point are presented in Table 2. Only two clusters from the SF-36 (physical health and emotional health) included all of the items present in the corresponding SF-36 scale. The correlation of bowel symptoms from the UCLA-PCI bowel function scale was <0.4 and therefore not strong enough to form a cluster. It is clear from the analysis that symptom clusters are not always the same as the scales of the UCLA-PCI or SF-36. The urinary function cluster consisted of three (urinary leak, urinary control and dripping/wetting) out of the five UCLA-PCI urinary function scale symptoms. The remaining two symptoms (number of pads and urinary leak interfering with sex) did not exhibit high enough correlation with the three symptoms to be included in the urinary function cluster (<0.2 and <0.3, respectively).

Symptom clusters were evaluated separately for each time point and were very similar at each point, so relatively stable over time. In addition, there were core symptoms that were always present in a cluster across time. They are marked in bold in Table 2. For example, the sexual function cluster had five core symptoms (erection ability, orgasm ability, quality of erections, frequency of erections and sexual function overall) and two that were present in the cluster intermittently (sexual desire and awakened with erections). There was one symptom in the sexual function scale of UCLA-PCI (intercourse) that was not correlated enough to belong to the sexual function cluster at any time point.

### Longitudinal Profiles of Symptom Clusters: Trajectory of Treatment and Recovery

**Fig 1:**
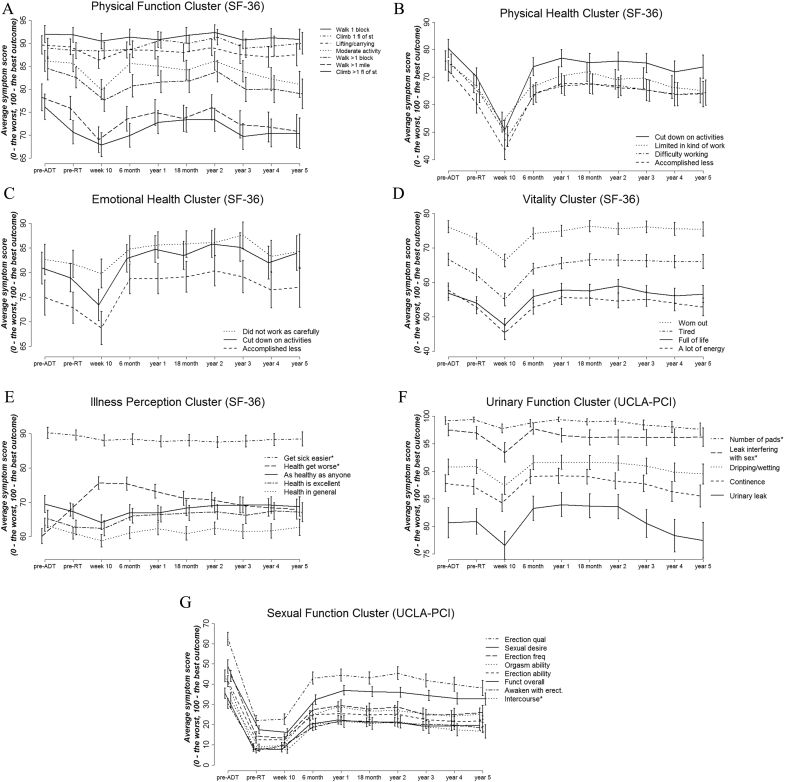
Longitudinal profiles of symptom clusters: (A) physical function; (B) physical health; (C) emotional health; (D) vitality; (E) illness perception; (F) urinary function: (G) sexual function. (A)–(E) are items from the 36 item Short-Form Health Survey (SF-36); (F) and (G) are items from the University of California, Los Angeles Prostate Cancer Index (UCLA-PCI).

The longitudinal profiles of symptoms over the 5 years of follow-up are presented in Figure 1. They document the trajectory of treatment and recovery after radiotherapy for patients with PCa. From these time profiles, we observe an increase in symptom intensity by week 10, which is represented by a peak fall in scores. This decrease in function and HRQOL clearly corresponds to the onset of acute symptoms due to radiotherapy. In relation to that, we can distinguish two types of trend. The first, where the onset of acute symptoms starts before radiotherapy, is during the period when men receive ADT. Clusters such as physical health, vitality and illness perception are examples of this type of decline in HRQOL. However, the functional decline due to ADT is also very prominent for the sexual function cluster. We observe that the sexual function of patients drops dramatically after ADT as compared with the baseline levels, and for many men it does not return to baseline levels even after many years post-treatment.

The second type of acute functional and HRQOL decline due to treatment can be observed for the physical function, emotional health and urinary function clusters. They are relatively stable during ADT and the acute decline is mainly due to radiotherapy. For these clusters, symptoms and HRQOL recover by 6 months after radiotherapy, reaching higher than the pretreatment levels. In addition to acute symptoms, for some symptom clusters we can also observe an increase in late symptoms. This decline in function and HRQOL starts after year 2 post-radiotherapy. This is especially prominent for the physical function, emotional health and urinary function clusters.

### Risk Factors and Antecedents for Late Symptom Clusters: Regression Analysis

The results of regression analysis are presented in Table 3. Older age was associated with decreased long-term function and HRQOL for physical function, physical health and sexual function clusters (*P* < 0.001). For all symptom clusters, baseline and acute scores (week 10) were identified as significant antecedents of late symptoms at year 3. This was represented by the high variation between patients (*P* < 0.001) in all seven models. Patients with more severe baseline symptoms had more severe acute and late symptoms. The radiotherapy dose had no statistically significant effect on the severity of symptom clusters over time (*P* = 1.000) in all seven models. However, decreased physical function and physical health, as well as illness perception and vitality clusters, contributed to the increased severity of symptom clusters over time and were significant risk factors for late symptoms at year 3.

**Table 3:** Multivariate mixed effects analyses show the contribution of potential risk factors to the changes in profiles of symptom clusters over time. A separate model was obtained for each of the symptom clusters: physical function, physical health, emotional health, vitality, illness perception, urinary function and sexual function. Random effects included radiotherapy dose, time and individual patient variation. Fixed effects included baseline patient characteristics, medical history and other symptom clusters that were used as independent variables (potential risk factors). Independent variables with preliminary significant associations of *P* < 0.05 were retained in the final regression models. A statistical significance level was set at *P* < 0.01

Dependent variable	Independent variable	Regression coefficient	Standard error	95% confidence interval		*P*-value
Physical function cluster	Age (10 years)	–3.64	1.10	–5.8	–1.49	0.001
	Hypertension (no)	3.69	1.41	0.93	6.44	0.009
	Physical health	0.16	0.02	0.13	0.19	<0.001
	Vitality	0.22	0.04	0.15	0.29	<0.001
	Illness perception	0.23	0.03	0.18	0.28	<0.001
	Urinary function	0.06	0.03	0.01	0.11	0.014
Physical health cluster	Age (10 years)	–5.61	1.18	–7.93	–3.29	<0.001
	Physical function	0.26	0.03	0.21	0.31	<0.001
	Emotional health	0.34	0.02	0.3	0.38	<0.001
	Vitality	0.81	0.04	0.74	0.88	<0.001
Emotional health cluster	Physical health	0.37	0.02	0.32	0.41	<0.001
	Vitality	0.31	0.05	0.21	0.40	<0.001
	Illness perception	0.12	0.03	0.05	0.19	0.001
	Urinary function	0.11	0.03	0.04	0.17	0.001
	Sexual function	–0.05	0.02	–0.10	–0.01	0.034
Vitality cluster	Age (10 years)	2.61	0.68	1.29	3.95	<0.001
	Physical function	0.08	0.01	0.05	0.10	<0.001
	Physical health	0.18	0.01	0.17	0.21	<0.001
	Emotional health	0.07	0.01	0.05	0.09	<0.001
	Illness perception	0.26	0.02	0.22	0.29	<0.001
	Urinary function	0.05	0.02	0.02	0.08	0.002
	Sexual function	0.06	0.01	0.04	0.08	<0.001
Illness perception cluster	Age (10 years)	3.13	1.02	1.11	5.11	0.002
	Stage	–1.25	0.51	–2.25	–0.26	0.014
	Gleason score	1.22	0.47	0.31	2.13	0.009
	Hypertension (no)	4.73	1.31	2.17	7.30	<0.001
	Inflammatory bowel (no)	7.06	3.12	0.96	13.17	0.024
	Physical function	0.14	0.02	0.10	0.18	<0.001
	Vitality	0.49	0.03	0.44	0.54	<0.001
Urinary function cluster	Gleason score	1.24	0.49	0.29	2.20	0.011
	Emotional health	0.04	0.01	0.02	0.07	0.002
	Vitality	0.12	0.03	0.07	0.18	<0.001
	Sexual function	0.05	0.02	0.02	0.08	0.003
Sexual function cluster	Age (10 years)	–11.95	1.24	–14.38	–9.52	<0.001
	Diabetes (no)	7.46	2.96	1.66	13.26	0.012
	Vitality	0.25	0.04	0.18	0.32	<0.001
	illness perception	0.10	0.03	0.04	0.17	0.001
	urinary function	0.08	0.03	0.02	0.14	0.008

## Discussion

We studied the trajectory of symptom clusters before, during and after radiotherapy for PCa, by analysing symptom clusters over 5 years after radiotherapy. This study contributes to the limited body of research documenting symptom clusters in PCa and radical radiotherapy [37] as well as identifying important targets for improving patient outcomes. The symptom clusters identified were different from those presented in the literature so far [15], [16]. We did not find the correlation of fatigue and mental health symptoms with PCa-specific symptoms (urinary, bowel or sexual) observed by Maliski *et al.* [15]. However, vitality and emotional health clusters were significant risk factors for the urinary and sexual function clusters, as shown by regression analysis. Capp *et al.* [16] focused on rectal toxicity in a longitudinal study. However, we did not observe a cluster associated with bowel dysfunction. The difference in the composition of symptom clusters between different studies may be due to the clustering approach or PROs tools used, or due to the differing treatments or population characteristics [38].

The composition of symptom clusters was different from that of PROs scales. Some clusters did not form and some of the items present in a scale were excluded from the cluster. For example, number of pads and urinary leak interfering with sex are the two items of the urinary function scale (UCLA-PCI) not included in the urinary function cluster. We observe from Figure 1F that both of these symptoms are rarely reported by patients (their longitudinal profiles occupy the top part of the graph) and any true impact from these symptoms would be difficult to detect. Symptom clustering enables identification of groups of correlated symptoms that are more prevalent and more relevant to patients. Therefore, it allows recognition of symptom clusters specific to the type of cancer and its treatment, which is important for appropriate symptom management [39], [40]. Clinical practice often focuses on single symptoms [41], [42]. However, the association and interaction between cancer symptoms should be explored [43], [44], [45]. In addition to the clinical consideration of symptoms in groups, symptom clusters allow a flexible and sample-specific way of analysing PROs [46]. Multiple-item scales should be revised prior to statistical analysis and clinical utilisation of PROs. The use of cumulative scores based on symptom clusters rather than scales was shown to be a better predictor of late symptoms [47].

**Fig 2:**
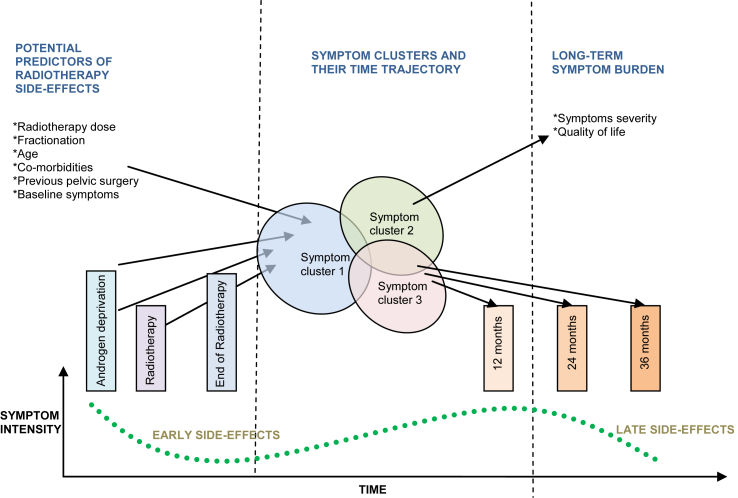
The time trajectory of symptom clusters during neoadjuvant androgen deprivation therapy and radiotherapy treatment and 5 years of follow-up. The role of symptom clusters and the mechanism of late radiotherapy morbidity including consequential late effects and other potential risk factors influencing acute and late symptoms and radiotherapy-related toxicity.

Symptom clusters were relatively stable over time, with core symptoms always present in the cluster. The stability of symptom clusters across the time trajectory has also been shown in other longitudinal studies [48], [49]. However, this study is unique as there is no other study that reports PCa symptom clusters for as long as 5 years. Studies describe sentinel symptoms, which can be used as indicators of symptom clusters in clinical assessment [50], [51]. This cannot only be used in identifying patients at high risk of long-term symptoms, but core cluster symptoms have also been recognised as targets for symptom management interventions [52]. These findings are important and can lead to targeted prehabilitation approaches in the form of lifestyle interventions before ADT and radiotherapy. ADT reduces muscle mass and strength, so it affects physical and functional health [8], [53]. The National Institute for Health and Care Excellence (NICE) recommends that men on ADT receive 12 weeks of exercise intervention to reduce fatigue symptoms [54]. A recent systematic review and meta-analysis suggests that this is also beneficial for lower body strength and aerobic fitness [55]. Figure 2 illustrates potential risk factors that can contribute to increased radiotherapy side-effects and may influence symptom severity and reduce HRQOL. The effectiveness of physical rehabilitation has been shown to decrease cancer and treatment-related morbidity and improve late radiotherapy outcomes [56], [57], [58].

Baseline and acute (end of treatment) symptom clusters were associated with decreased long-term function and HRQOL in this population. This was independent of the radiotherapy dose and added to the evidence towards consequential late effects [18], [19]. Physical health and physical function clusters were important antecedents of symptoms at 3 years in older men. Older men are likely to have multiple underlying health problems and this may influence their physical and functional health [21], [22]. Screening of health status and the management of comorbidities is important before treatment as this may improve fitness. This is recommended in the International Society of Geriatric Oncology (SIOG) guidance for older cancer patients [59]. Poorer fitness may be a consequence of an individual's poorer health rather than chronological age alone. There is evidence that even a small increase in physical activity, such as walking (5 h moderate intensity physical activity per week) can significantly reduce PCa-specific mortality in men with low-risk tumours [60], but this may not be the same in metastatic PCa [55].

Previous analysis of PROs from the MRC RT01 trial identified that gastrointestinal toxicity increased from baseline for up to 3 years after radiotherapy [61]. Acute bladder symptoms were also a precursor of late symptoms and patients with pretreatment bladder symptoms were more likely to report bladder symptom at 5 years [62]. Emerging evidence around phosphodiesterase type 5 (PDE5) inhibitors suggests that these agents may have an impact not only on sexual but also on urinary symptoms [63], [64]. However, the clinical mechanisms of this effect remain unclear. Provision of support in the cancer recovery pathway, through survivorship plans, has mixed results in terms of benefits, but referral to voluntary sector services could improve self-management and early symptom support [65]. This reflects findings from other studies [66], [67], [68] and underpins the importance of using a broader PRO assessment that includes symptoms, function and HRQOL measurements. If it is established that pretreatment PRO scores predict poor post-radiotherapy outcomes then there is a need to intervene and show through research that the consequences of cancer treatment can be prevented. Future work is needed to establish whether poor PROs and thus men's long-term quality of life can be improved.

### Strengths and Limitations

The strength of this study is that MRC RT01 is a large dataset with a long follow-up. PROs of PCa patients are recorded for 5 years after treatment and, unusually, the trial asked participants to complete multiple PROs. These data allowed us to observe the late deterioration in symptoms and HRQOL that starts to emerge from year 2 after radiotherapy. This deterioration, possibly due to radiation fibrosis, deserves attention and engagement of early management strategies [69]. With the long-term follow-up we were also able to establish the effect of early outcomes on late PROs. These findings are important in the identification of patients at risk of late radiotherapy-related toxicity. The limitation of this study is the large number of trial participants not returning PROs at certain time points and the fact that clustering analysis and longitudinal profiles are presented for different numbers of patients at each point in time. In addition, UCLA-PCI is now an old tool that has largely been replaced in PCa health assessment by the Expanded Prostate Cancer Index Composite (EPIC) instrument [70]. However, because EPIC adapts similar scoring rules for its items, investigating symptom clusters over time can be beneficial.

## Conclusions

This study uniquely contributes to the understanding of the consequences of cancer treatment and how symptom clusters can be used in supportive care of men before and after PCa radiotherapy. Early symptoms, older age, physical function and physical health were associated with the severity of late symptoms. Therefore, early management of age-related comorbidities and prehabilitation of physical and functional status by promoting physical activity, as well as guiding patients to support and counselling services, should complement treatment planning to aid recovery during and after radiotherapy. It has been shown here and in other PCa studies that age and pretreatment health and function could be used to identify patients at greater risk of post-treatment symptoms [71]. Subgroups of patients who are likely to have poorer functional and HRQOL outcomes can be identified using PROs. This has been undertaken in women with breast cancer [72]. Early assessment using PROs and patient stratification that incorporates risk factors may help to identify men who require prehabilitation and additional support throughout their treatment and recovery. Furthermore, there is a need for more research studies that investigate the impact of personalised interventions to improve symptoms and long-term outcomes of radiotherapy patients.

## Conflict of Interest

Professor David Dearnaley, Consultant Clinical Oncologist, London, has attended, and received honoraria, for advisory boards and served as a consultant for Takeda, Amgen, Astellas, Sandoz and Janssen Pharma. Abiraterone acetate was developed at the Institute of Cancer Research, which therefore has a commercial interest in the development of this agent. Professor David Dearnaley is on the Institute's Rewards to Inventors list for abiraterone acetate.

## References

[1] Yeruva S.L.H., Nwabudike S.M., Ogbonna O.H., Oneal P. (2015). Aromatase inhibitor-induced erythrocytosis in a patient undergoing hormonal treatment for breast cancer. Case Rep Hematol.

[2] Maddams J., Utley M., Moller H. (2012). Projections of cancer prevalence in the United Kingdom, 2010–2040. Br J Cancer.

[3] Globocan (2012). http://www.globocan.iarc.fr/Pages/fact_sheets_population.aspx.

[4] Yip K., McConnell H., Alonzi R., Maher J. (2015). Using routinely collected data to stratify prostate cancer patients into phases of care in the United Kingdom: implications for resource allocation and the cancer survivorship programme. Br J Cancer.

[5] Andreyev H.J.N., Wotherspoon A., Denham J.W., Hauer-Jensen M. (2010). Defining pelvic-radiation disease for the survivorship era. Lancet Oncol.

[6] Carlsson S., Drevin L., Loeb S., Widmark A., Lissbrant I.F., Robinson D. (2016). Population-based study of long-term functional outcomes after prostate cancer treatment. BJU Int.

[7] Dal Pra A., Cury F.L., Souhami L. (2010). Combining radiation therapy and androgen deprivation for localized prostate cancer—a critical review. Curr Oncol.

[8] Nguyen P.L., Alibhai S.M., Basaria S., D'Amico A.V., Kantoff P.W., Keating N.L. (2015). Adverse effects of androgen deprivation therapy and strategies to mitigate them. Eur Urol.

[9] Kerns S.L., Ostrer H., Rosenstein B.S. (2014). Radiogenomics: using genetics to identify cancer patients at risk for development of adverse effects following radiotherapy. Cancer Disc.

[10] Miaskowski C., Dodd M., Lee K. (2004). Symptom clusters: the new frontier in symptom management research. J Natl Cancer Inst Monogr.

[11] Xiao C. (2010). The state of science in the study of cancer symptom clusters. Eur J Oncol Nurs.

[12] Barsevick A.M. (2007). The elusive concept of the symptom cluster. Oncol Nurs Forum.

[13] Ferreira K.A., Kimura M., Teixeira M.J., Mendoza T.R., da Nobrega J.C., Graziani S.R. (2008). Impact of cancer-related symptom synergisms on health-related quality of life and performance status. J Pain Symptom Manag.

[14] Fan G., Filipczak L., Chow E. (2007). Symptom clusters in cancer patients: a review of the literature. Curr Oncol.

[15] Maliski S.L., Kwan L., Elashoff D., Litwin M.S. (2008). Symptom clusters related to treatment for prostate cancer. Oncol Nurs Forum.

[16] Capp A., Inostroza-Ponta M., Bill D., Moscato P., Lai C., Christie D. (2009). Is there more than one proctitis syndrome? A revisitation using data from the TROG 96.01 trial. Radiother Oncol.

[17] Knapp K., Cooper B., Koetters T., Cataldo J., Dhruva A., Paul S.M. (2012). Trajectories and predictors of symptom occurrence, severity, and distress in prostate cancer patients undergoing radiation therapy. J Pain Symptom Manag.

[18] Peach M.S., Showalter T.N., Ohri N. (2015). Systematic review of the relationship between acute and late gastrointestinal toxicity after radiotherapy for prostate cancer. Prostate Cancer.

[19] Pinkawa M., Holy R., Piroth M.D., Fischedick K., Schaar S., Székely-Orbán D. (2010). Consequential late effects after radiotherapy for prostate cancer – a prospective longitudinal quality of life study. Radiat Oncol.

[20] Dearnaley D.P., Jovic G., Syndikus I., Khoo V., Cowan R.A., Graham J.D. (2014). Escalated-dose versus control-dose conformal radiotherapy for prostate cancer: long-term results from the MRC RT01 randomised controlled trial. Lancet Oncol.

[21] Smith A.W., Reeve B.B., Bellizzi K.M., Harlan L.C., Klabunde C.N., Amsellem M. (2008). Cancer, comorbidities, and health-related quality of life of older adults. Health Care Financ Rev.

[22] Sogaard M., Thomsen R.W., Bossen K.S., Sorensen H.T., Norgaard M. (2013). The impact of comorbidity on cancer survival: a review. Clin Epidemiol.

[23] Posternak V., Dunn L.B., Dhruva A., Paul S.M., Luce J., Mastick J. (2016). Differences in demographic, clinical, and symptom characteristics and quality of life outcomes among oncology patients with different types of pain. Pain.

[24] Skerman H.M., Yates P.M., Battistutta D. (2009). Multivariate methods to identify cancer-related symptom clusters. Res Nurs Health.

[25] Aktas A., Walsh D., Hu B. (2014). Cancer symptom clusters: an exploratory analysis of eight statistical techniques. J Pain Symptom Manag.

[26] Dearnaley D.P., Sydes M.R., Graham J.D., Aird E.G., Bottomley D., Cowan R.A. (2007). Escalated-dose versus standard-dose conformal radiotherapy in prostate cancer: first results from the MRC RT01 randomised controlled trial. Lancet Oncol.

[27] Dearnaley D.P., Sydes M.R., Langley R.E., Graham J.D., Huddart R.A., Syndikus I. (2007). The early toxicity of escalated versus standard dose conformal radiotherapy with neo-adjuvant androgen suppression for patients with localised prostate cancer: results from the MRC RT01 trial (ISRCTN47772397). Radiother Oncol.

[28] Sydes M.R., Stephens R.J., Moore A.R., Aird E.G., Bidmead A.M., Fallowfield L.J. (2004). Implementing the UK Medical Research Council (MRC) RT01 trial (ISRCTN 47772397): methods and practicalities of a randomised controlled trial of conformal radiotherapy in men with localised prostate cancer. Radiother Oncol.

[29] Litwin M.S., Hays R.D., Fink A., Ganz P.A., Leake B., Brook R.H. (1998). The UCLA Prostate Cancer Index: development, reliability, and validity of a health-related quality of life measure. Med Care.

[30] Ware J.E., Sherbourne C.D. (1992). The MOS 36-item short-form health survey (SF-36). I. Conceptual framework and item selection. Med Care.

[31] Little R.J.A., Rubin D.B. (2002). Statistical analysis with missing data.

[32] Sterne J.A.C., White I.R., Carlin J.B., Spratt M., Royston P., Kenward M.G. (2009). Multiple imputation for missing data in epidemiological and clinical research: potential and pitfalls. BMJ.

[33] Litwin M.S. (1994). UCLA-PCI including the RAND SF-36 v2 health-related quality of life scoring instructions. https://eprovide.mapi-trust.org/instruments/ucla-prostate-cancer-index/scoring.

[34] Corey D.M., Dunlap W.P., Burke M.J. (1998). Averaging correlations: expected values and bias in combined Pearson rs and Fisher's z transformations. J Gen Psychol.

[35] Zelefsky M.J., Levin E.J., Hunt M., Yamada Y., Shippy A.M., Jackson A. (2008). Incidence of late rectal and urinary toxicities after three-dimensional conformal radiotherapy and intensity-modulated radiotherapy for localized prostate cancer. Int J Radiat Oncol Biol Phys.

[36] Luke S.G. (2017). Evaluating significance in linear mixed-effects models in R. Behav Res Meth.

[37] Thavarajah N., Chen E., Bedard G., Lauzon N., Zhou M., Chu D. (2012). Symptom clusters in patients with prostate cancer: a literature review. J Pain Manag.

[38] Kirkova J., Walsh D. (2007). Cancer symptom clusters – a dynamic construct. Support Care Cancer.

[39] Kwekkeboom K.L. (2016). Cancer symptom cluster management. Semin Oncol Nurs.

[40] Skerman H.M., Yates P.M., Battistutta D. (2012). Cancer-related symptom clusters for symptom management in outpatients after commencing adjuvant chemotherapy, at 6 months, and 12 months. Support Care Cancer.

[41] Vij A., Kowalkowski M.A., Hart T., Goltz H.H., Hoffman D.J., Knight S.J. (2013). Symptom management strategies for men with early-stage prostate cancer: results from the Prostate Cancer Patient Education Program (PC PEP). J Cancer Educ.

[42] Skolarus T.A., Ragnoni J.A., Garlinghouse C., Schafenacker A., Webster D., Hager P. (2017). Multilingual self-management resources for prostate cancer survivors and their partners: results of a long-term academic-state health department partnership to promote survivorship care. Urology.

[43] Barsevick A.M., Whitmer K., Nail L.M., Beck S.L., Dudley W.N. (2006). Symptom cluster research: conceptual, design, measurement, and analysis issues. J Pain Symptom Manag.

[44] Chen M.L., Lin C.C. (2007). Cancer symptom clusters: a validation study. J Pain Symptom Manag.

[45] Miaskowski C., Aouizerat B.E. (2007). Is there a biological basis for the clustering of symptoms?. Semin Oncol Nurs.

[46] Lemanska A., Chen T., Dearnaley D.P., Jena R., Sydes M.R., Faithfull S. (2017). Symptom clusters for revising scale membership in the analysis of prostate cancer patient reported outcome measures: a secondary data analysis of the Medical Research Council RT01 trial (ISCRTN47772397). Qual Life Res.

[47] Lemanska A., Cox A., Kirkby N.F., Chen T., Faithfull S. (2014). Predictive modelling of patient reported radiotherapy-related toxicity by the application of symptom clustering and autoregression. Int J Stat Med Res.

[48] Gift A.G., Stommel M., Jablonski A., Given W. (2003). A cluster of symptoms over time in patients with lung cancer. Nurs Res.

[49] Kim H.-J., Barsevick A.M., Tulman L., McDermott P.A. (2009). Treatment-related symptom clusters in breast cancer: a secondary analysis. J Pain Symptom Manag.

[50] Aktas A. (2013). Cancer symptom clusters: current concepts and controversies. Curr Opin Support Palliat Care.

[51] Brown J.K., Cooley M.E., Chernecky C., Sarna L. (2011). A symptom cluster and sentinel symptom experienced by women with lung cancer. Oncol Nurs Forum.

[52] Barsevick A.M. (2007). The concept of symptom cluster. Semin Oncol Nurs.

[53] Chang D., Joseph D.J., Ebert M.A., Galvao D.A., Taaffe D.R., Denham J.W. (2014). Effect of androgen deprivation therapy on muscle attenuation in men with prostate cancer. J Med Imaging Radiat Oncol.

[54] NICE (2008). Prostate cancer: diagnosis and management. http://www.nice.org.uk/guidance/cg175.

[55] Bourke L., Smith D., Steed L., Hooper R., Carter A., Catto J. (2016). Exercise for men with prostate cancer: a systematic review and meta-analysis. Eur Urol.

[56] Silver J.K., Baima J. (2013). Cancer prehabilitation: an opportunity to decrease treatment-related morbidity, increase cancer treatment options, and improve physical and psychological health outcomes. Am J Phys Med Rehabil.

[57] Shun S.-C. (2016). Cancer prehabilitation for patients starting from active treatment to surveillance. Asia-Pacific J Oncol Nurs.

[58] Silver J.K. (2015). Cancer prehabilitation and its role in improving health outcomes and reducing health care costs. Semin Oncol Nurs.

[59] Droz J.-P., Aapro M., Balducci L., Boyle H., Van den Broeck T., Cathcart P. (2014). Management of prostate cancer in older patients: updated recommendations of a working group of the International Society of Geriatric Oncology. Lancet Oncol.

[60] Wang Y., Jacobs E.J., Gapstur S.M., Maliniak M.L., Gansler T., McCullough M.L. (2017). Recreational physical activity in relation to prostate cancer-specific mortality among men with nonmetastatic prostate cancer. Eur Urol.

[61] Syndikus I., Morgan R.C., Sydes M.R., Graham J.D., Dearnaley D.P. (2010). Late gastrointestinal toxicity after dose-escalated conformal radiotherapy for early prostate cancer: results from the UK Medical Research Council RT01 Trial (ISRCTN47772397). Int J Radiat Oncol Biol Phys.

[62] Barnett G.C., De Meerleer G., Gulliford S.L., Sydes M.R., Elliott R.M., Dearnaley D.P. (2011). The impact of clinical factors on the development of late radiation toxicity: results from the Medical Research Council RT01 trial (ISRCTN47772397). Clin Oncol.

[63] Mahmood J., Shamah A.A., Creed T.M., Pavlovic R., Matsui H., Kimura M. (2016). Radiation-induced erectile dysfunction: recent advances and future directions. Adv Radiat Oncol.

[64] Chughtai B., Ali A., Dunphy C., Kaplan S.A. (2015). Effect of phosphodiesterase inhibitors in the bladder. Asian J Urol.

[65] McCabe M.S., Faithfull S., Makin W., Wengstrom Y. (2013). Survivorship programs and care planning. Cancer.

[66] Braithwaite D., Satariano W.A., Sternfeld B., Hiatt R.A., Ganz P.A., Kerlikowske K. (2010). Long-term prognostic role of functional limitations among women with breast cancer. JNCI.

[67] Demark-Wahnefried W., Pinto B.M., Gritz E.R. (2006). Promoting health and physical function among cancer survivors: potential for prevention and questions that remain. J Clin Oncol.

[68] Brown J.C., Harhay M.O., Harhay M.N. (2015). Physical function as a prognostic biomarker among cancer survivors. Br J Cancer.

[69] Faithfull S., Lemanska A., Aslet P., Bhatt N., Coe J., Drudge-Coates L. (2015). Integrative review on the non-invasive management of lower urinary tract symptoms in men following treatments for pelvic malignancies. Int J Clin Pract.

[70] Wei J.T., Dunn R.L., Litwin M.S., Sandler H.M., Sanda M.G. (2000). Development and validation of the expanded prostate cancer index composite (EPIC) for comprehensive assessment of health-related quality of life in men with prostate cancer. Urology.

[71] Resnick M.J., Barocas D.A., Morgans A.K., Phillips S.E., Chen V.W., Cooperberg M.R. (2014). Contemporary prevalence of pretreatment urinary, sexual, hormonal, and bowel dysfunction: defining the population at risk for harms of prostate cancer treatment. Cancer.

[72] Dodd M.J., Cho M.H., Cooper B.A., Miaskowski C. (2010). The effect of symptom clusters on functional status and quality of life in women with breast cancer. Eur J Oncol Nurs.

